# Access and reimbursement pathways for digital health solutions and *in vitro* diagnostic devices: Current scenario and challenges

**DOI:** 10.3389/fmedt.2023.1101476

**Published:** 2023-02-20

**Authors:** Andrea Mantovani, Claudia Leopaldi, Cassandra Maria Nighswander, Rossella Di Bidino

**Affiliations:** ^1^ Alira Health, Milan, Italy; ^2^ Alira Health, Basel, Switzerland; ^3^ Fondazione Policlinico Universitario Agostino Gemelli IRCCS, Graduate School of Health Economics and Management (ALTEMS), Rome, Italy

**Keywords:** digital therapeutics, *in vitro* diagnostics, reimbursement, regulatory, access, software, device, digital health

## Abstract

**Objectives:**

Digital therapeutics (DTx) are innovative solutions that use meaningful data to provide evidence-based decisions for the prevention, treatment, and management of diseases. Particular attention is paid to software-based *in vitro* diagnostics (IVDs). With this point of view, a strong connection between DTx and IVDs is observed.

**Methods:**

We investigated the current regulatory scenarios and reimbursement approaches adopted for DTx and IVDs. The initial assumption was that countries apply different regulations for the access to the market and adopt different reimbursement systems for both DTx and IVDs. The analysis was limited to the US, European countries (Germany, France, and UK), and Australia due to maturity in digital health product adoption and regulatory processes, and recent regulations related to IVDs. The final aim was to provide a general comparative overview and identify those aspects that should be better addressed to support the adoption and commercialization of DTx and IVDs.

**Results:**

Many countries regulate DTx as medical devices or software integrated with a medical device, and some have a more specific pathway than others. Australia has more specific regulations classifying software used in IVD. Some EU countries are adopting similar processes to the Digital Health Applications (DiGA) under Germany's Digitale-Versorgung Gesetz (DVG) law, which deems DTx eligible for reimbursement during the fast access pathway. France is working on a fast-track system to make DTx available to patients and reimbursable by the public system. The US retains some coverage through private insurance, federal and state programs like Medicaid and Veterans Affairs, and out-of-pocket spending. The updated Medical Devices Regulation (MDR) and *In Vitro* Diagnostic Regulation (IVDR) in the EU includes a classification system specifying how software integrated with medical devices, and IVDs specifically must be regulated.

**Conclusion:**

The outlook for DTx and IVDs is changing as they are becoming more technologically advanced, and some countries are adapting their device classifications depending on specific features. Our analysis showed the complexity of the issue demonstrating how fragmented are regulatory systems for DTx and IVDs. Differences emerged in terms of definitions, terminology, requested evidence, payment approaches and the overall reimbursement landscape. The complexity is expected to have a direct impact on the commercialization of and access to DTx and IVDs. In this scenario, willingness to pay of different stakeholders is a key theme.

## 1. Introduction

The World Health Organization (WHO) recently released the global strategy on digital health as a visionary document that provides a framework for countries to implement and expand digital health services. The vision of the strategy is to improve for everyone and everywhere the access to appropriate, accessible, affordable, scalable, and sustainable person-centric digital health solutions to prevent, detect and respond to epidemics and pandemics. The strategy is to support the development of infrastructures and applications that enable countries to use health data to promote health and well-being. The aim of the WHO's Global Strategy on Digital Health 2020–2025 is to develop partnerships at the national, regional and global levels to align resources and investments to focus on sustainability and growth of digital health (1).

To reach these goals the regulation related to the access to the market and criteria adopted for reimbursement play a crucial role.

As recognized by the WHO, digital health solutions could help in disease detection. Key technologies for disease detection are *In Vitro* Diagnostic Devices (IVDs). They are used in clinical, laboratory or outpatient settings with the aim specifically to help in the detection of diseases and, as a consequence, in the selection of appropriate treatment protocols. Nowadays, the use of digital technology in healthcare is a way to increase access to diagnostic and triage services and may improve the quality of this process (2). Emerging solutions including the use of artificial intelligence, health bots, online triage systems may present opportunities for patient care and address issues of high costs and time consumption (2).

Therefore, more attention must be paid to software. Under that point of view a strong connection among Digital Therapeutics (DTx) and IVDs emerged.

For those reasons our analysis focused on a specific subset of health technologies, (DTx and IVD) and on a specific phase of their cycle of life (access to the market and reimbursement).

## 2. Background

### 2.1. Digital therapeutics (DTx)

Digital health is an umbrella term that encompasses a variety of terms, including e-health, m-health and telehealth and captures everything from electronic patient records, remote monitoring, connected devices, digital therapeutics and more.

Despite disagreement in the definition of digital health (3), it is clearly how the digitalization of health is associated with a long list of aspects from appropriate management of big data to reliability of Artificial Intelligence (AI)/Machine Learning (ML) algorithms. Not secondary, it is the need to guarantee information security and allow to patient real control over their own health data.

The global digital health market was expected to reach US$ 881 Billion in 2021, with a CAGR (Compound Annual Growth Rate) of 20.14% in 2027 (4). Growing smartphone adoption, better Internet connectivity, improved healthcare information technology (IT) infrastructure, an increase in the prevalence of chronic diseases, a rising demand for remote patient monitoring services, and easier access to virtual healthcare, will all contribute to the market growth.

Not all digital health technologies have the same clinical value. We decided to focus our attention on Digital Therapeutics (DTx), which are evidence-based therapeutic interventions driven by software to prevent, manage, or treat a medical disorder or disease (5). DTx are patient-facing software applications that have a proven clinical benefit. For example, DTx can support patients in self-managing symptoms and thereby improve their quality of life and other clinical endpoints. Digital Therapeutics use digital implements, such as mobile devices, apps, sensors, virtual reality, the internet of things, and other tools to spur behavioral changes in patients. So far, about 250 different products have been identified with about 150 of these being commercially available (6). Digital therapeutic development can have a positive impact on providing well customized health services as their design is tailored to fit patients’ needs.

Digital Therapeutics are either as a standalone therapy or in conjunction with more conventional treatments, such as pharmacological or in-person therapy or with certain hardware or other sensory or mechanical devices. The treatment depends on the collection and processing of digital measurements. Because of the digital nature of the methodology, data is collected and analyzed as both a progress report and a preventative measure. Currently, treatments are developed for the prevention and management of a wide variety of diseases and conditions, such as type II diabetes (7), congestive heart failure (8), Alzheimer's disease (9), anxiety, depression (10), and several others.

Taken together, this patient-centered disruption is rapidly changing how the industry operates and how services are developed and delivered, rebuilding relationships between key stakeholders such as the research-based industry, patients, healthcare professionals, health institutions, and regulators. The tools and technologies that will reshape healthcare are rapidly becoming available in hospitals and homes across Europe. The transition to a more digital hospital infrastructure requires investments from people, technological platforms, and processes. If working together properly, the integration of digital technologies can result in higher quality care, improved operational efficiencies, and increased patient satisfaction (11).

#### 2.1.1. DTx regulations and reimbursement pathways

Given their potential role in the delivery of care, the use of DTx should be proven through clinical trial studies to demonstrate their clinical efficacy and should be evaluated by Health Technology Assessment (HTA) bodies in order to be reimbursed and prescribed by physicians, as it happens with medicinal products.

Our study moved from a double assumption: countries differ in their regulatory systems for digital health solutions and therefore for DTx, in addition these systems are not yet fully prepared to regulate DTx, and all other health technologies associated with them, as IVDs.

Indeed, currently, countries have an applicable framework for digital solutions, they are included under the regulatory framework for medical devices, software as a medical device (SaMD), or software in a medical device (SiMD). Products within these frameworks are categorized into classes based on their risk classification. Occasionally, in DTx products are included in these classifications depending on a countries’ regulations. Countries differ in their guidelines to allow the product to be recognized for approval by regulatory bodies. Some countries may not have any established guidelines that fit DTx products and reimbursement may be unlikely due to a lack of incentive.

For each country in scope (US, Germany, France, UK, and Australia), the regulatory status is outlined and preceded by a brief description of the institutions involved. Relevant reimbursement opportunities in terms of public coverage, private coverage, employer-sponsored, and consumer-funded healthcare are identified. This provides a setting for the challenges and opportunities that will be mentioned later in this manuscript.

### 2.2. *In vitro* diagnostic devices (IVDS)

*In vitro* diagnostics are recognized as a diagnostic test that is done on blood or tissue samples extracted from the human body to detect diseases or give an overall view of a patient's health condition. They are typically used in laboratories, but some forms can be used in one's home. For instance, direct-to-consumer (DTC) IVDs are diagnostic tests that require the consumer to collect a specimen to send to a laboratory for analysis to assess one's risk for developing a disease.

Regardless of the setting of use, there are different types of IVDs that can be used depending on the patient's condition or what type of treatment they are likely to benefit from. Next-generation sequencing tests are useful tools to utilize precision medicine in treating patients for diseases, while Companion Diagnostics (CDx) are IVDs that are used in combination with a therapeutic drug and are strictly link to the identification of biomarkers. For instance, CDx could help to identify the appropriate patient group most likely to benefit from a therapeutic product, to predict serious adverse reactions, or to monitor the response to treatment in order to improve the dosage scheme (12).

Disruptive diagnostics is a term used to describe innovative technologies able to make diagnostic products more effective, efficient, and accurate (13). It describes many forms of diagnostic tests, such as genome sequencing and imaging techniques, but more recently has included Artificial Intelligence or Machine Learning based diagnostic tools. These specific diagnostics use Al algorithms known as deep learning to completely interpret information from large amounts of data. New technologies utilizing AI are being developed for many indications to increase efficiency, reduce time to treatment, lower costs, and allow for more precise and effective therapies. In genomic testing diagnostics, deep learning is be used to identify cancer cells, determine their type, and predict what mutations may occur in a tumor from images of a specific cancer (14).

#### 2.2.1. *In vitro* diagnostic regulations and reimbursement pathways

The WHO uses three strategic priorities to emphasize the importance of having IVDs available to those who need them. These priorities include access to quality, affordable, and appropriate healthcare products to advance universal health coverage, address health emergencies, and promote healthier populations. The WHO prequalification team evaluates the safety and performance of tests according to international standards so they can be eligible for procurement. This can give countries the opportunity to determine if they should purchase certain diagnostics. In addition, the WHO maintains a list of IVD recommended at the point of care that should be used in every country, the WHO Model List of Essential *In Vitro* Diagnostics (EDL). The IVDs on this list are endorsed by the latest clinical evidence to support their use everywhere. Developments of IVD regulations depend significantly on how medical devices are classified. These regulations, as well as those of digital health solutions are subject to adaptation, because of the advancements within the world of medical technology (15).

## 3. Aim of the analysis

We investigated the current regulatory scenarios and reimbursement approaches adopted for DTx and IVDs. The starting assumption was that countries apply different regulations for the access to the market and adopt different reimbursement systems for both DTx and IVDs.

Our specific focus was on the international context where digital approaches (software) to disease detection are becoming quite common. The analysis was limited to the US, European countries (Germany, France, and UK), and Australia due to maturity in digital health product adoption and regulatory processes, and recent regulations related to IVDs. Nonetheless their market relevance was taken into account.

Our attention, as stated, was concentrated on the regulatory and reimbursement processes. With the term regulatory we referred to the process that must be followed to access to the market, while with reimbursement processes, we meant steps and criteria followed to define the price reimbursed by a public or private institution.

The final aim was to provide a general comparative overview and identify those aspects that should be better addressed to support the adoption and commercialization of DTx and IVDs.

## 4. Methods

A scoping review of publications and grey literature was conducted to understand the current regulatory scenarios and reimbursement approaches adopted for DTx and IVDs.

First, grey literature review was conducted for DTx to understand the current level of usage, acceptability, reimbursement, and assessment. The review included the U.S. Food and Drug Administration (FDA), the European Medicines Agency (EMA) and main Health Technology Assessment (HTA) agencies public reports. Included HTA agencies were: National Institute for Health and Care Excellence (NICE) for United Kingdom, Haute Autorité de Santé (HAS) for France, Gemeinsame Bundesausschuss (G-BA) for Germany, and the Pharmaceutical Benefits Advisory Committee (PBAC) based in Australia.

For the scoping review on IVDs, an analysis was done of the regulatory guidelines for IVDs in each country. Updated laws and pathways were covered to assess the present and future landscape of usage of IVDs in healthcare practices. In appropriate situations, the regulation of Software in IVDs was assessed to show the acceptability of digital maturity in certain countries.

In addition, one important source was the DTx Alliance (DTA), a non-profit trade association of industry leaders and stakeholders dedicated to providing information on the background, current access status, and value of DTx products in select countries. It is the leading international organization on digital therapeutic thought leadership and education.

In-depth information was uncovered and helped facilitate a comparison across the countries in scope considering the pathways for DTx, gaps in access, and future prospects for DTx integration. The specific categories of information assessed from Digital Therapeutics Alliance (DTA) were product category, regulatory agency involved, product risk classifications, regulatory review, pre-submission opportunities, regulatory guidelines, and product recognition. The same process was performed for reimbursement opportunities, which included information on public and private insurance coverage, employer-sponsored healthcare, and consumer funding status. The most relevant types of coverage were reported.

## 5. Results

In this section, we provide a brief description of the agencies and national bodies involved in regulatory and reimbursement decisions that are relevant for DTx and IVDs. The purpose is to give a general overview of the bodies involved in the entire adoption and commercialization process of a product.

Data reported in Tables 1, 2 is the base of our analysis.

**Table 1:** Agencies involved in the regulation and reimbursement of medical technology.

Country	Main regulatory agency	Reimbursement/HTA agency
USA	The FDA is responsible for approving new DTx within the software-as-a-medical-device category.	Depends on the classification of the device and the level of coverage requested (Medicare, Medicare Advantage, Medicaid, the Department of Defense)
Germany	The Federal Institute for Drugs and Medical Devices (BfArM) and The German Institute of Medical Documentation and Information (DIMDI)	Institute for the Hospital Remuneration System (InEK)
France	National Agency for the safety of Medicines and Health Products (ANSM)	National Commission for Evaluation of Medical Devices and Health Technologies (CNEDiMTS)
United Kingdom	Medicines and Healthcare products Regulatory Agency (MHRA)	National Institute for Health and Care Excellence (NICE); local commissioning groups (CCGs)
Australia	Therapeutic Goods Administration (TGA) for medical devices	The Medical Services Advisory Committee makes recommendations on inclusions in Medical Benefits Schedule (MBS)

**Table 2:** Documents containing guidelines for the regulation of digital therapeutics and *in vitro* diagnostics.

Country	Guidelines to be met for digital therapeutics	Guidelines to be met for *in vitro* diagnostics
US	Title 21 of the Code of Federal Regulations (21 CFR)	
Germany	Medical Device Regulation (MDR) (16)	*In Vitro* Diagnostic Regulation (IVDR) (16)
France		
UK	The Medical Technologies Evaluation Program (MTEP)	The Medical Device Regulations 2002 (17)
Australia	How the TGA regulates software-based medical devices (18)	Australian regulatory guidelines for medical devices (ARGMD) (19); Principles of *In Vitro* Diagnostic (IVD) Medical Devices Classification

In Table 1 the main regulatory and reimbursement agencies for medical technology for each country are specified to introduce the bodies involved in these processes.

Table 2 lists the relevant documents that contains existing guidelines DTx and IVDs must follow to be regulated in given countries.

In the following, more details are reported for each selected country. In addition, when investigating the specific national scenario, reimbursement options are specified given that both digital therapeutic and *in vitro* diagnostic devices may be reimbursed through public or private insurance options or other solutions (as out-of-pocket payments or employee sponsored healthcare (20).

### 5.1. DTx regulatory status

#### 5.1.1. US

The FDA is responsible for approving new DTx within the software-as-a-medical-device (SaMD) category. It is explicitly recognized that they are developed and validated differently than traditional medical devices (defined as hardware-based MD).

FDA supports various plans to advance digital health technology approvals, including the Software Precertification (Pre-Cert) Pilot Program launched in July 2017 and concluded in September 2022 (21). This program is expected to inform the development of a future regulatory model that provides more streamlined and efficient oversight of software-based medical devices. One of the most relevant conclusions of the pilot is the emerged need of an “appropriate new legislative authority… to support the development and implementation of a new regulatory paradigm”, while FDA will continue to develop policies and tools within the current authorities. That the main reason of the creation of the Digital Health Center of Excellence, whose mission is meanwhile to provide advice and support for the regulatory review of digital health technology.

Strictly related are the regulations and guidelines defined by:

• The Health Insurance Portability and Accountability Act (HIPAA), which protects the privacy and security of certain health information.
• The Federal Trade Commission (FTC) Act, which prohibits deceptive or unfair acts, including false or misleading claims about safety and performance of apps.
• The FTC’s Health Breach Notification Rule which requires certain businesses to provide notifications following breaches of personal health record information (22). These guidelines are applicable under all circumstances.

For medical devices, including SaMD, the US has a specific process in which the product is classified by risk, either as:

• Class I (general controls)
• Class II (special controls in addition to general controls)
• Class III (premarket approval in addition to general controls)

Some Class I and II products are exempt from marketing submission but must still register and list with the FDA.

Some following pathways for regulatory review are the 510(k) pathway, which is a pre-market submission for medical devices to be approved by the FDA, the *De Novo* pathway, which requires a market authorization to demonstrate assurance of safety and efficacy, and the Pre-market Approval (PMA) pathway, which is a stringent market submission to demonstrate safety and effectiveness.

A clinical trial is generally required for the PMA pathway. As product recognition, Class I and II exempt are FDA coded only if a product code exists and enforcement discretion products are not FDA listed. Class II products are FDA cleared under the 510(k) pathway, and FDA granted under the *De Novo* pathway. Class III products are FDA approved under the PMA pathway (22).

Another pathway that is voluntary for some medical devices and device-led combination products the Breakthrough Devices Program. This has replaced the Expedited Access Pathway and Priority Review for medical devices and helps accelerate the market approval of products that can be effective for life-threatening or debilitating conditions.

#### 5.1.2. EU member states

At the European level, the regulation, (EU) 2017/745, is a regulation of the European Union on the clinical investigation and sale of medical devices for human use. No specific legal regulation exists on DTx while the EMA and the European Commission (EC) are beginning to explore these solutions. Compared with the US, Europe appears as a fragmented with no single harmonized process for national body approval and reimbursement. It is a hard-to-crack market for DTx innovators, even with new regulations such as MDR (EU Regulation 2017/745). Some European countries have their own unique way of assessing digital health, meaning that they have a lower reward than the bigger markets (i.e., the USA).

However, the European Commission (EC) is proposing the European Health Data Space, for digital transformation in all EU member states, including regulations for data privacy, EU General Data Protection Regulation, medical devices, AI, and HTA (23). This is an ecosystem comprised of health-related rules and regulations, standards, and a common framework to support the emergence of digital health practices. This has the goal of empowering individuals through the use of digital health and the control of personal data. This action will aim to increase trust in systems handling personal data, and foster innovation in policy making and regulatory activities.

At a national level, some European countries, such as Germany and France use a consistent framework for classifying products for regulatory review. The DTx manufacturer must first complete a self-assessment risk clarification in accordance with the standards defined by the Medical Device Coordination Group (MDCG) to determine the level of CE mark necessary for the product. The framework comprises Class IIa, which includes most DTx software, and Class IIb, which includes DTx products with higher risks or consequences. Class I DTx do not qualify for regulatory review and remain under MDR. For product recognition, countries in the EU require a CE mark (20).

#### 5.1.3. UK

According to the DTA, UK still regulates DTx products under the MDR to determines the necessary CE mark. The types of products that qualify for regulatory review remain the same as those in France and Germany. UKCA mark requirements will be updated soon, however they are still derived from the EU CE Mark regulations. Product recognition with a CE Mark will remain this way until July 2023 (20).

For software and AI-based medical devices, the MHRA has announced a new program, the Software and AI as Medical Device Change Programme to emphasize the inclusion of these types of devices in regulatory processes. The Change Programme will identify the necessary steps for a regulatory framework to protect these DTx and other software-based medical devices. The UK government has highlighted some key points to describe their approach in the implementation of this program. With the expansion of AI and software in medical devices, a priority of this program is to cover possible regulations to ensure these devices are used safely and responsibly. A deeper focus on patient engagement and communities will also be a key priority as the purpose is for these devices to follow the proper regulation and reach users successfully (24).

Work packages (WP) were established to understand the problems that needed to be addressed in order to move forward with a successful regulatory framework including software and AI-based medical devices WP1 covers the qualification of SaMD and software in medical devices to establish a clear definition of these types of products according to these updated regulations. Specific guidance will be published to understand certain scenarios that bring uncertainties when regulating medical devices with a software component:

• SaMD versus wellbeing and lifestyle software products
• Medical device software versus IVD software
• Research use only “exemption” For SaMD
• Custom made devices
• Requirements for software in a kit/software system/software in procedure pack/software as a service
• Accessories to a medical device or IVD

Other WPs cover topics including Classification, Premarket requirements, Post market surveillance, Cyber Secure Medical Devices, AI Rigor, AI interpretability, and AI Adaptivity. The overall purpose of these WPs is to ensure safe adoption and use and protect patients while simultaneously accelerating innovation (24).

#### 5.1.4. Australia

The Therapeutic Goods Administration is responsible for regulating DTx in Australia. There is a specific framework for regulating software-based medical devices. Any regulated product that meets the definition of a medical device, and is not excluded or exempt, is required to be listed in the Australian Register of Therapeutic Goods (ARTG). If a device is excluded or exempt, it means the TGA lacks some authority over the regulation of this device, and therefore does not have to be listed. There are three steps for a device to be placed on the ARTG:

1. The manufacturer must obtain a certification from the TGA or a comparable oversees regulator. This is only required for manufacturers of class II and above medical devices.
2. The sponsor of the medical device must submit the manufacturer’s certificate to the TGA before submitting and ARTG application
3. The sponsor must then submit the ARTG inclusion application

A pre-submission process is available for any TGA submission and can be requested at any stage of the submission process, with the proper documentation present (marketing documents, data, instruction manuals).

All DTx products must comply with the Essential Principles, with various relating to SaMD. All other Essential Principles that apply to the product must be considered. Clinical evidence is not necessarily required, however, many innovative, class II, and class III products may require an RCT (20).

### 5.2. DTx reimbursement pathways

#### 5.2.1. Summary

Reimbursement options for DTx vary among countries discussed. Coverage may be available situationally (e.g., therapeutic area) or due to the type of insurance scheme. Table 3 summaries the implications of the current scenario in terms of coverage of DTx to highlight the possibilities and limitations present in each country. Coverage possibilities analyzed include public and private insurance schemes, employer sponsored coverage, and consumer funded coverage.

**Table 3:** Reimbursement landscape in given countries.

Country	Public health insurance	Private health insurance	Employer sponsored	Consumer funded
US	Products covered depending on insurance scheme	Most products covered, with some limitations	Coverage available through direct negotiation	Out-of-pocket payments possible
Germany	Statutory health insurance coverage; Digital health product directory (DiGA); selective contracts	Not obliged to cover products but possible through direct negotiation	Not common	Low willingness to pay out-of-pocket
France	Individual funding decisions; Some experimental coverage options	Some coverage of specific items	Not common	Low willingness to pay out-of-pocket
UK	No national reimbursement, local organizations for funding and reimbursement	Some products partially covered, but not a common route	Not common	Low willingness to pay out-of-pocket
Australia	Some schemes available depending on therapeutic area	Some products covered depending on benefit and efficiency gains	Protection insurance providers may fund digital health	Difficult; co-pays common

#### 5.2.2. US

Regarding reimbursement in the US, there are different possibilities depending on the classification of the device and the level of coverage requested.

With public coverage, there are different subsets of government funded insurances that apply to digital health solutions. These include Medicare, Medicare Advantage, Medicaid, and coverage by the Department of Defense. Medicaid care plans may consider fee-for-service coverage or benefit-program coverage on a state-by-state basis. The Department of Defense covers some DTx products that have a hardware component.

With private insurance, some products are covered through a fee-for-service basis. Exempt DTx medical devices and enforcement discretion classified medical devices are reimbursed through a set of unique device identifier codes. Regulated Class II and III medical devices may or may not require a prescription (20).

#### 5.2.3. Germany

On 19 December 2019, the Digital Supply Act (DVG) came into force in Germany. This meant that since 2020, clinicians have been able to prescribe, and Statutory Health Insurance (SHI) funds reimburse medical apps and digital health treatments (DiGA—BfArM). To ensure such treatments are available to patients as quickly as possible, the regulatory agency, Bundesinstitut für Arzneimittel und Medizinprodukte (BfArM), has developed a new process to speed up reimbursement: the DiGA Fast-Track. Germany has established pathways for the reimbursement of DTx through the DiGA pathway and selective contracts with SHIs. Products listed on the DiGA directory are fully reimbursed by SHIs. The BfArM is responsible for decisions regarding DiGA listings.

The BfArM assesses these DiGA treatments in terms of patient benefit, data protection and information security and quality. After an application is received, the BfArM has 3 months to check whether the products meet the requirements set out in the DiGA Ordinance. However, the manufacturers must also prove that the app has a positive effect on patient care. If the assessment is successful, the product can then be included in the DiGA list. The products on this list can then be prescribed and are covered by SHI funds (25).

To be included in the DiGA directory, products must meet the following specific requirements:

• be a Class I or IIa medical device, compliant with Annex I and II of DiGAV on data protection, IT security, interoperability, robustness, consumer protection, ease of use, support of healthcare providers, quality of medical content and patient safety
• prove at least one healthcare benefit positive Versorgungseffekte) which could be a medical benefit or patientenrelevante Struktur- und Verfahrensverbesserungen
• demonstrate clinical relevance in a retrospective study, preferably a randomized control trial. It is possible to have a permanent listing where all requirements are filled or a preliminary listing where there are 12 months for clinical evidence to be submitted. Selective contracts allow for full or partial reimbursement from negotiating with individual SHIs to deciding on reimbursement of individual SHIs (25).

#### 5.2.4. France

When the value of the DTx has been assessed, the second step is the negotiation of the price. Currently, the actors involved are the company and the CEPS (26).

While for DTx which are not eligible to the standard procedure or in the absence of robust demonstration of their clinical benefit/risk profile, other pathways are possible, as in the case of telemonitoring experimentation.

In 2023 a new regulation for reimbursement of DTx is expected. Agence du Numérique en Santé, the HAS (Haute autorité de santé) and the ANSM (Agence nationale de sécurité du medicament et des produits de santé), are developing guidelines taking as reference the German experience (27).

To be eligible for reimbursement in France and funded through the standard P&R procedure regarding medical devices, DTx will need to provide:

• A CE marking as a medical device and privacy
• Compliance with EU General Data Protection Regulation
• A General Health Technology Assessment carried out by the Commission nationale d’evaluation des dispostifs médicaux et des technologies de (CNEDIMT) and HAS
• An actual medical benefit assessment
• A Clinical Evidence Evaluation
• A demonstration of clinical and socio-economic added value
• However, up to now, there are no current reimbursement pathways for DTx products, but it is possible to make individual funding decisions (28).

Firstly, the CNEDIMTs gives an opinion on the actual medical benefit, a Service attendu (“SA”). The opinion about SA determines whether the connected medical device is reimbursed (SA sufficient) and the rate of its reimbursement by the French national health insurance (important SA: 65% reimbursement, moderate: 30%; low: 15%) or not (insufficient medical benefit). The SA must take into consideration: severity of the disease, efficacy/adverse effects ratio, intended role in the therapeutic strategy in comparison with other available interventions, public health benefits. To be eligible for reimbursement, the DTx must demonstrate a sufficient efficacy/safety ratio in a robust clinical study. The HAS published guidelines to explain how to develop a digital therapeutic which complies with the French HTA requirements and which elements are needed to be favorably assessed by the CNEDIMTs (28).

If the SA is evaluated sufficient, the CNEDIMTs gives its opinion as well on the “Added Medical benefit assessment” [America Society of Anesthesiologist (ASA)]. The added medical benefit measures the digital therapeutic added clinical value compared with existing interventions already reimbursed and is used to determine the price. The digital therapeutic is innovative when the assessment is from ASA I to III. If the new digital healthcare product brings a minor improvement compared to the current strategy, the assessment is an ASA IV. In case of no improvements of the clinical value compared with other alternatives, the assessment is an ASA V (28).

#### 5.2.5. UK

Funding and reimbursement of DTx in the UK is done on a local level by NHS organizations. There are 43 integrated care systems (ICSs) responsible for DTx funding. Digital Therapeutics and other digital health products are included in the national NHS library if they fulfill the necessary criteria in the Digital Technology Assessment Criteria (DTAC) framework. However, this does not imply reimbursement (29). Evidence demonstrating the safety and efficacy of the device will be necessary to prove it is worth being funded. There is an alternative approach through a separate ring-fenced budget, however, this route may hinder the ability of HCPs to provide the best treatment pathways for patients and discourage the integration of DHTs into treatment pathways.

The NHS has identified three key criteria that DHTs must meet in order to receive funding:

1. Be appropriately CE/UKCA marked to be placed on the market in the UK, ensuring that the device is safe, and works as described by the manufacturer
2. Pass the DTAC to ensure that they meet core standards for clinical safety, data protection, security, interoperability, accessibility, and usability; and
3. Be recommended by NICE, to ensure they are plausibly cost-effective and therefore represent value for the NHS (30)

The purpose of having these criteria in place is to create a high standard and set an example for other countries to follow. Having standards to follow can also promote innovation by having clear access and reimbursement mechanisms in place to follow.

#### 5.2.6. Australia

Reimbursement for DTx through public health insurance may be possible in certain circumstances depending on the therapeutic area, if the digital product is in companion to a device, prosthetic, therapeutic, or service. Schemes include:

• Pharmaceutical Benefits Scheme (PBS)
• Medical Benefits Schedule (MBS)
• National Diabetes Services Scheme (NDSS)
• National Disability Insurance (NDIS)

Although there is no direct pathway for coverage of DTx through private insurance, some insurers may decide to engage if the product reduces cost of care, creates efficiency gains for the health insurer, or increases engagement (31).

### 5.3. IVD regulatory status

#### 5.3.1. US

The FDA classifies IVDs as medical devices into Class I, II, or III according to its risk to assure safety and effectiveness in practice. It is possible for an IVD to be classified as a biological product, which would be subject to section 351 of the Public Health Service Act. An early classification of the IVD will determine which regulatory path to take (32). The classification of existing IVDs can be found in the Code of Federal Regulations list, specifically in 21 CFR 862, 21 CFR 864, 21 CFR 866.

Like other medical devices, IVDs are subject to premarket and post-market controls. There are a few relevant processes conducted by the FDA to assess the quality of the product and the relevance of any related issues.

A pre-submission process is encouraged by the FDA in some circumstances in order for the submitter to receive feedback. Some circumstances include:

• If the device involves a new technology, intended use, or analyte, so the FDA can be informed on the use of this novel feature
• If there is assistance needed in defining regulatory pathways
• If the study involves complex data
• If the predicate or reference method is unclear or uncertain
• If the new device is a multiplex device capable of simultaneously testing a large number of analytes (33)

A 510(k) pathway review process for IVDs evaluates the analytical performance of the device compared to the predicate including measures like bias or inaccuracy of the new device, the imprecision of the new device, and the analytical specificity and sensitivity.

A premarket approval (PMA) is a process where the FDA evaluates the safety and effectiveness of Class III. For IVDs specifically, the safety is linked the impact of the device performance on the patients’ health, specifically the what the impact would be from a false negative or false positive.

New guidelines have been developed to re-assess the inclusion of Laboratory Developed Tests (LDTs) as IVDs. The FDA issued a draft guidance and a discussion paper that outlines regulatory requirements and limitations surrounding certain types of LDTs (34).

#### 5.3.2. EU member states

The *In Vitro* Diagnostic Regulation (IVDR) is the new regulatory basis for placing on the market, making available, and putting into service *in vitro* diagnostic medical devices on the European market and the EU's current directive on *in vitro* diagnostic medical devices which will be replaced (98/79/EC). As a European regulation, it will be effective in all EU member states and European Free Trade Commission (EFTA) states immediately without need to be transferred into the law of the respective states; however, national laws may be adapted to support some requirements in more detail.

The IVDR became effective on May 25, 2017. The EC are implementing several guidance documents to satisfy the requirements to be met.

Patient safety is central in the new regulation. Various requirements for manufacturers are imposed to demonstrate that the medical device is safe and performs consistently as intended when it is used as intended by the manufacturer. The current directive dates from 1998. It is now quite old and no longer fit for purpose. The introduction of the new directives brings a more robust set of requirements, which are much more adaptable to the change over time.

With the start of application, some in-vitro diagnostic devices fall into higher levels and must be declared compliant for the first time with the involvement of a Notified Body. Due to the now phased introduction [Regulation (EU) 2022/112], the transition periods for all IVD apply as follows:

• For products that already required the involvement of a Notified Body under the IVDR, 26 May 2025 is the key deadline. For devices that are newly classified by the IVDR and now require the involvement of a Notified Body for the first time, the following deadlines apply regarding the transition periods:
  ○ 05.2025—Class D
  ○ 05.2026—Class C
  ○ 05.2027—Class B
  ○ 05.2027—Sterile Class A

Until the above-mentioned deadlines, products can be placed on the market with a declaration of conformity according to 98/79/EC. However, the declaration of conformity must be issued before 26 May 2022 and there may not be any significant changes to the design and purpose of the medical devices before the end of the transition period. The phased introduction of the IVDR means no change compared to the previous requirements of the IVDR for *in vitro* diagnostic medical devices that do not require the involvement of a Notified Body. For them, the IVDR will be fully applicable from 26 May 2022. This concerns the following:

Class A non-sterile devices

• “new” *in vitro* diagnostic medical devices for which no certificate or declaration of conformity has yet been issued in accordance with Directive 98/79/EC (IVDD).

Manufacturers of IVD may still have well-filled warehouses. Therefore, the question may arise on how to proceed with the remaining goods. All products can be placed on the market in accordance with the cut-off date rules mentioned at the beginning of this chapter; However, placing the remaining goods on the market under Directive 98/79/EC (IVDD) is no longer possible. Manufacturers need to implement different strategies to handle IVD stocks (23).

Medical Device Software (MDSW), as intended by the manufacturer to be used with an IVD, is treated specifically as a IVD MDSW (23). Specific classifications of this subset of medical devices are highlighted in Table 4.

**Table 4:** MDSW classification under the IVDR.

Type of software/intended purposed	Regulation
Software intended to be installed on a fully automated enzyme-linked immunosorbent assay (ELISA) analyzer, and intended to determine the Human HbA1c concentration in serum from the results obtained with a Human HbA1c ELISA, intended to screen for and diagnose diabetes and monitor diabetic patients	Class C per Rule 3 (k)
Software within a PAP stain automated cervical cytology screening system, intended to classify the PAP cervical smear as either normal or suspicious	Class C per Rule 3 (h)
Software for the interpretation of automated readings of line immunoassay for the confirmation and determination of antibodies to HIV-1, HIV-1 group O and HIV-2 in human serum and plasma	Class D per Rule 1
Software that uses maternal parameters such as age, concentration of serum markers and information obtained through fetal ultrasound examination for evaluating the risk of trisomy 21	Class C per Rule 3 (1)

In addition to the change in classification rules, there is an increased harmony between the IVDR and the MDR the equivalent for medical devices, including a focus on clinical evaluation and increased transparency through the wider supply chain.

#### 5.3.3. UK

Regulations for IVDs are established in the UK MDR 2002. This document gives general guidance on different products considered for approval, the classification of different IVDs, and the following steps to take once the item is classified.

There are four existing categories which define the risk that the IVD may pose in a healthcare setting. These categories include:

• General IVDS
• IVDs for self-testing
• IVDs in the classifications stated in Part IV of the UK MDR 2002, Annex II List B
• IVDs in the classifications stated in Part IV of the UK MDR 2002, Annex II List A

A conformity assessment must be followed according to the category the IVD falls under. Certain requirements must be met to ensure compliance with designated standards (17).

#### 5.3.4. Australia

IVDs are regulated based on the risk they pose in a therapeutic setting. The framework that applies to all medical devices applies to IVDS as well and includes:

• Pre-market assessment—conformity assessment
• Market authorization—inclusion in the ARTG
• And post-market monitoring—continuing compliance with all regulatory, safety and performance requirements and standards.

IVDs have a separate risk-classification including:

• Class 1—No public health risk or low personal risk
• Class 2—Low public health risk or moderate personal risk
• Class 3—Moderate public health risk or high personal risk
• Class 4—High public health risk

There is a framework available that lists the types of IVD software that is regulated (31). The five circumstances where IVD software is regulated are outlined in Table 5.

**Table 5:** IVD software regulations in Australia.

Type of software/intended purposed	Regulation
1. Software that is built into an IVD medical device	The instrument with the installed/embedded software is a single IVD. The IVD classification rule 1.6 in Schedule 2A specifies that all instruments that are IVDs are Class I IVDs
2. Software that is supplied separately to and IVD medical device but is intended to operate or influence the IVD	A distinct IVD that is separate to the IVD instrument or analyzer. According to Regulation 3.3 (5) under *Principles for applying the classification rules* in the Regulations, software that drives or influences a medical device has the same classification as the medical device
3. Software that is intended to be used to provide diagnostic or therapeutic information and is not intended to drive or influence an IVD medical device	An IVD. IVD software that is not intended to drive or influence an IVD instrument (or medical device that is not an IVD) is classified according to its intended purpose. This applies to Class II, III, and IV IVDs
4. Software that is intended for handling patient-related information. Including results from IVD devices, which may be used in combination with an IVD instrument	Does not meet the definition of a therapeutic good. Software that handles clinical information and has a diagnostic and therapeutic function is considered an IVD. Software that performs a manipulation on the data that affects the interpretation of results, or generates new diagnostic data, makes the software a medical device
5. Corrections to software errors	A correction to the IVD (not a distinct IVD). Correction to software may be a product correction under the Uniform Recall Procedure for Therapeutic Goods. If the functionality that changes the intended purpose of the software is added, the software may be considered a distinct IVD to the original

Adapted from software as *in vitro* medical devices (IVDs), therapeutic goods administration (TGA), 2013. Copyright 2013 by TGA.

### 5.4. IVD reimbursement pathways

#### 5.4.1. US

Considering the reimbursement of IVDs and LDTs in the US, Medicare has specific processes for billing, coding, and pricing that are administered by the Centers for Medicare and Medicaid Services (CMS). These decisions also act as a baseline for private insurance companies.

The CMS relies on a network of Medicare administrative contractors to serve as contact points for healthcare providers and the Medicare Fee for Service program. Laboratory tests are reimbursed by the CMS under the Clinical Laboratory Fee Schedule (CLFS) which defines reimbursement rates for each type of test. To ensure that reimbursement codes are issued, updated, and maintained as they should be, Current Procedural Terminology (CPT) codes are used. The CPT codes are five-character codes that indicate what type of care has been provided.

For LDTs specifically, Category I CPT codes should be reviewed to describe the specific diagnostic test. A Medicare Administrative Contractor (MAC) and private payers are asked to provide reimbursement. In addition, proprietary laboratory analyses (PLA) codes are assigned to specific tests defined in the Protecting Access to Medicare Act (PAMA). These include advanced diagnostic laboratory tests (ADLTs), clinical diagnostic laboratory tests (CDLTs), multianalyte assays with algorithmic analyses (MAAA) and genomic sequencing procedures (GSPs) (35).

#### 5.4.2. European countries

In most of the European countries, IVD tests that are performed at the hospital, are included in drug related group (DRG) tariff and laboratories are funded using a global budget principle. However, in out-patient settings, many countries have specific reimbursement frameworks for IVD tests, most commonly using a fee-for-service payment model. Table 6 lists existing situations for obtaining reimbursement in Germany, France, and the UK.

**Table 6:** Reimbursement solutions for IVDs in selected European countries.

Country	Health system	Benefit basket	Catalog characteristics	Elements of tariff calculation
Germany	Social Health Insurance	Uniform value scale	Positive list, generic description of medical act	Non-medical acts involved/time spent based on expert opinion, material costs, investment costs
France	Etatist Social Health Insurance	Nomenclature of the medical biological acts	Positive list, generic description of medical act	Time spent on medical act, stress level during medical act, mental effort, technical competence
UK	National Health Service	Benefit basket as defined by the National Institute for Health and Care Excellence guidance on technologies	Commercial tests are specified within NICE guidance	Cost parameters in relation to efficiency, appropriateness, and quality; standard costs of related medical acts in the regions

Adapted from shedding light on reimbursement policies of companion diagnostics in European countries p. 608. Copyright 2020 by ScienceDirect.

#### 5.4.3. Australia

Private insurance is fairly common for patients in Australia to utilize, with about 44% of the population using private hospital cover in 2018 (36). Patients can have private insurance for hospital or general cover. While private insurance can cover a portion of the associated costs, the remainder is covered by the Medicare Benefits Schedule or MBS.

The Medicare Benefits Schedule is a list of services which includes diagnostic tests that the Australian government subsidizes.

## 6. Discussion

Digital therapeutics are likely to become a critical player in healthcare in coming years. Self-regulating alone is no longer the only available option. Government and regulatory agencies are working on the definition of *ad hoc* regulatory pathways able to respond to the specific features of DTx and to their pace of technological change.

Our analysis showed the complexity of the issue demonstrating the fragmentation of regulatory systems for DTx and IVDs at international level. Despite limiting our attention to few countries, differences emerged in terms of definitions, terminology, requested evidence, payment approaches and the overall reimbursement landscape. In the majority of the selected countries, regulatory pathways for DTx are aligned to those already adopted for medical devices even in terms in requested evidence to submit to regulatory agencies. Even if the definition of DTx is not included in the definition of a medical device, they are considered a specific kind of medical device as in the case of FDA, which defines them as SaMD.

The scenario is evolving, however, as demonstrated by the creation in US of a specific unit inside FDA dedicated to DTx and the conclusions of the Pre-Cert Pilot Program. It clearly concluded that a new legislative authority could be necessary in the near future. The German DiGA is very specific in terms of the evidence that needs to be submitted for accessing the market. Some countries have shown interest in working to develop a similar Framework (37). France has a conditional reimbursement pathway, PECA (38). This supports a product for reimbursement while the evidence generation gap is being filled. This pathway covers remote monitoring solutions where there is no “generic line” for the specific indication, as well as DTx. To be covered, there needs to be some promise that an evidence gap will be closed, and a clinical benefit will be meaningful (38).

These conclusions could be extended to IVD. With this, countries are recognizing these technological developments, and fitting them into national regulations. It is important to note that the new regulation distinguishes among a software that is intended to be used, alone or in combination with a medical device or IVDR. Australia, as noted earlier, also has a specific guidance on how to classify types of IVDs that involve a type of software. The US has established some acceptance of software use in a proposal from 2019, to establish the foundation for pre-market review for AI and machine learning software modifications. This, however, does not specify how to handle the integration of this with IVDs (39).

The complexity is expected to have a direct impact on the commercialization of and access to DTx and IVDs. In this scenario, willingness to pay of different stakeholders is a key theme as well as willingness to consider as part of reimbursement packages.

### 6.1. Accessibility to DTx

In European countries, approval and reimbursement are decided separately, slowing down the accessibility of DTx products. In the US, public and private providers have different approaches to reimbursement. Private payers are much more ahead in their willingness to pay for DTx products. Medicare, as one of the public healthcare providers, has no mechanism for reimbursing DTx. Since there is no broad system and DTx products may be categorized at different levels due to differing mechanisms and benefits, they are evaluated by payers on an *ad-hoc* basis. In the US, since each DTx product is not reimbursed based on specific criteria, it could be reimbursed using a few different approaches. These include out-of-pocket payments by patients, as a medical device where the app is sold to a hospital based on its medical benefit, or *via* a health savings account (20).

Europe has quite advanced public reimbursement systems in comparison to the US, with more established reimbursement methods; however, inconsistencies among countries makes it difficult to accommodate all DTx products, IVD-related included. Some European countries may be ahead in terms of reimbursement. For example, Germany is setting the stage for advanced, fast-tracked methods for reimbursement through the DiGA pathway for digital health apps, and some countries, such as France are trying to adopt similar methods. However, there are still challenges regarding reimbursement opportunities that limit scalability, given that regulatory frameworks differ among countries and that the regulatory framework is being standardized in the EU Member States, with the UK no longer in the EU.

There are some issues that are inhibiting the process of adopting a more common system for reimbursement within and among countries. It is slow-growing, and payment models vary greatly. Specific barriers to adopting this broader system include differing requirements, approaches, and frameworks at local and regional levels. Some issues with costs and low profitability for providers or unclear reimbursement of services associated with adopting DTx products do not give enough incentives for payers to support these instead of in-person medical visits. Some providers may also be hesitant in adopting some DTx solutions if data inputs are not coming from a standardized system. Health records may be disconnected from the hospital setting to DTx apps; thus, it can be difficult to measure efficacy over time. While reimbursement pathways are starting to look more promising in some ways, for example the US is developing digital formularies for broader claims reimbursement, challenges within the market remain (40).

There has been a step in the right direction with different bodies and organizations providing some detailed guidelines that use a common language on the navigation of DTx opportunities. The Digital Therapeutics Alliance lays out all necessary assessments DTx developers would need to follow to secure an optimal reimbursement pathway. This framework can be built on with any continuing developments. A similar scheme, IMPACT virtual care first (V1C) initiative, co-developed by the Digital Medicine Society (DiMe) offers various resources, contract guidelines, payment models, and a library of reimbursable codes (40).

Marketing tactics are a solution in promoting the use of DTx. If patients experience high satisfaction rates, this could potentially steer healthcare professionals in the direction of supporting them and getting the attention of policy makers and payers.

### 6.2. A look ahead

DTx developers still have a long way to go in navigating the feasible options regarding reimbursement for DTx products. To manage the risks with this and heighten opportunities, it is important to focus on the most promising therapeutic areas.

Communicating with payers and regulators early in the process can eliminate unnecessary steps in setting prices as well as provide a more realistic outlook by studying the most practical DTx solutions. Understanding where the DTx product is better suited geographically can also lead to its success, by focusing on the US for profitability and Europe for feasibility.

The new EU MDR should help to simplify the exchange of data on medical devices and improve data collection and post-market surveillance to reinforce end-user confidence in DTx solutions. However, Class I products have been upgraded and now require notified bodies designated by EU member states to assess them before a CE mark is granted; previously manufacturers provided self-declaration for these products. The requirements for pre- and post-market clinical data have increased and expert panels will now scrutinize all Class III and some Class IIb high-risk devices to ensure the safety and efficacy is supported by robust clinical data. This is envisaged to be a major hurdle for the access of these products as the number of notified bodies in Europe is limited. This may increase the data burden for DTx developers and potentially increase the approval time to market. A key objective of these regulatory changes is to ensure a high standard of safety and quality of digital health products while providing patients with quicker access and reimbursement to these innovative solutions. However, the EU MDR contains no specific provisions for DTx and further clarity on the subdivision into risk classes. The approach to be taken by notified bodies concerning regulations applicable to DTx would be useful and enable companies to determine the most appropriate route to market based on the risk–benefit each product brings.

With the MHRA introducing the Software and AI as a Medical Device Change Programme, regulation of DTx will hopefully become a more streamlined process that can promote similar activities in other countries. An emphasis on clinical evidence for DHT products that are on the way to approval and reimbursement will highly support their use and promote innovation in the world of medical devices. The specifics of this are available in the Evidence standards framework for digital health technologies. With this, the UK hopes to be a leading example in the regulatory environment for DHTs and be in line with the increasing adoption of software and AI solutions in healthcare settings. Involvement in the Digital Therapeutics Summit was an important opportunity to receive advice on how to tackle any challenges that remain for the outlook of DHTs in the UK (30).

### 6.3. The future for IVDs

The diagnostic landscape is ever changing, making healthcare practices cheaper, more effective, and more efficient. Disruptive diagnostics is a term to define using innovative technology to allow for more improved practices when detecting diseases. Many forms of disruptive diagnostics are using AI-supported technologies to improve the functionalities of diagnostic tests. These developments foster many positive changes in the healthcare landscape. More effective diagnostic tools allow for earlier detections and more efficient treatment plans. Personalized medicine will play a larger role as well, and hopefully lessen the burden on healthcare providers (13).

The US framework for regulating IVDs can be compared to the new IVDR in the EU. There are some notable differences in the type of regulatory oversight, classification method, and importance of clinical evidence. In the FDA framework, the recent development of reassessing how LDTs should be regulated has posed some uncertainties for manufacturers and laboratories. It is possible that a certain LDT is not considered and IVD anymore, so this leaves a need for updated regulations to include these LDTs as well (34).

Australia's framework for assessing software in combination with IVDs provides a benchmark for other countries to understand if this would be an effective element to implement. As it may be more common to see IVDs with a software component in the future, this could impact healthcare practices in terms of cost and efficiency.

## 7. Conclusion

In Europe, there are encouraging signs that countries are adopting more centralized pathways to regulate DTx, but further clarification is needed to drive future innovation and enhance patient access to a broader array of life-changing digital health solutions. Given that Germany has taken the lead on introducing a legal framework for certification of digital apps to achieve DTx status and reimbursement, it will be interesting to see what approaches other European countries implement over the coming months.

With regards to IVD, the IVDR is assisting in clearly classifying certain devices to assess whether they fit the criteria and can be regulated in certain countries. With some types of IVD adopting AI-supporting features, they must fit into these frameworks to be utilized in practice. If regulations are adapting to support these types of devices, there could be a promising outlook on disease detection being more efficient and effective. It is important that the technology-supported features are well regulated so they can work properly in the healthcare conditions they are addressing.
